# Supplementary material for: Seed Priming with Devosia sp. Cell-Free Supernatant (CFS) and Citrus Bioflavonoids Enhance Canola and Soybean Seed Germination
Source: Molecules. 2022 May 25;27(11):3410. doi: 10.3390/molecules27113410 (PMC9182190; doi:10.3390/molecules27113410)
Supplement: Supplementary file 1 [file molecules-27-03410-s001.zip › molecules-1692252-supplementary.pdf]

molecules

MDPI

Supplementary data

Seed Priming with *Devosia* sp. Cell-Free Supernatant (CFS) and Citrus Bioflavonoids Enhance Canola and Soybean Seed Germination

Ateeq Shah, Sowmyalakshmi Subramanian and Donald L. Smith *

Department of Plant Sciences, McGill University, Montreal, QC, Canada
Correspondence: Donald L. Smith; donald.smith@mcgill.ca

Supplementary data

Supplementary tables have been included to support and compliment the text, and to make the data accessible for readers observation (Tables S1 to S17)

"Flavopriming" and seed germination of canola and soybean (Tables)

The data in the Tables (S1 to S9) represent the mean percentage of 8 replications ± **standard** error. Different letters indicate values determined by Tukey's multiple mean comparison to be significantly different ($p < 0.05$) from each other.

Table S1. Canola seed germination as affected by flavonoid treatments (optimal conditions).

Treatments	18 h ± SE	24 h ± SE	30 h ± SE	36 h ± SE
p-value	0.0001	0.3700	0.4259	0.5290
Ctrl U.P (Unprimed)	85.00^{d} ± 2.83	93.75^{b} ± 2.27	95.00^{b} ± 1.89	95.63^{b} ± 1.48
Ctrl P (Primed)	90.00^{cd} ± 2.67	94.38^{b} ± 1.13	95.00^{b} ± 0.94	95.63^{b} ± 1.13
Fl 0.5 µL	95.00^{abc} ± 1.89	95.63^{ab} ± 1.99	97.50^{ab} ± 1.34	97.50^{ab} ± 1.34
Fl 1.0 µL	95.63^{ab} ± 1.48	95.63^{ab} ± 1.48	96.25^{ab} ± 1.57	96.88^{ab} ± 1.32
Fl 1.5 µL	98.13^{ab} ± 0.91	99.38^{a} ± 0.63	99.38^{a} ± 0.63	99.38^{a} ± 0.63
Fl 2.0 µL	97.50^{ab} ± 1.89	97.50^{ab} ± 1.89	98.13^{ab} ± 1.32	98.13^{ab} ± 1.32
Fl 2.5 µL	96.25^{ab} ± 1.83	97.50^{ab} ± 1.34	98.13^{ab} ± 1.32	98.75^{ab} ± 0.82
Fl 3.0 µL	96.25^{ab} ± 1.57	96.88^{ab} ± 1.32	96.88^{ab} ± 1.32	98.13^{ab} ± 1.32
Fl 3.5 µL	93.13^{bc} ± 2.66	95.00^{ab} ± 2.11	96.25^{ab} ± 1.83	97.50^{ab} ± 1.34
Fl 4.0 µL	98.75^{a} ± 1.25	98.13^{ab} ± 1.32	98.75^{ab} ± 0.82	98.75^{ab} ± 0.82
Fl 4.5 µL	96.88^{ab} ± 1.32	97.50^{ab} ± 0.94	97.50^{ab} ± 0.94	97.50^{ab} ± 0.94
Fl 5.0 µL	94.38^{abc} ± 1.48	96.88^{ab} ± 1.62	97.50^{ab} ± 1.64	97.50^{ab} ± 1.64

Table S2. Canola seed germination flavonoid treatments (125 mM NaCl conditions).

Treatments	18 h ± SE	24 h ± SE	30 h ± SE	36 h ± SE
p-value	< 0.0001	0.0185	0.0701	0.0870
Ctrl U.P (Unprimed)	26.88^{e} ± 2.66	90.63^{bc} ± 1.99	93.75^{abc} ± 1.83	94.38^{bc} ± 1.48
Ctrl P (Primed)	52.50^{d} ± 4.12	93.75^{ab} ± 1.83	95.63^{ab} ± 1.13	96.88^{abc} ± 1.32
Fl 0.5 µL	80.63^{bc} ± 4.77	91.25^{ab} ± 2.95	92.50^{bc} ± 2.50	93.75^{bc} ± 2.45
Fl 1.0 µL	75.63^{c} ± 8.04	84.38^{c} ± 4.95	90.63^{c} ± 2.90	94.38^{c} ± 1.99
Fl 1.5 µL	85.63^{abc} ± 6.44	96.88^{ab} ± 1.88	97.50^{a} ± 1.34	98.13^{ab} ± 1.32
Fl 2.0 µL	88.13^{ab} ± 2.98	95.63^{ab} ± 1.75	96.88^{ab} ± 1.32	97.50^{abc} ± 0.94
Fl 2.5 µL	88.13^{ab} ± 4.00	96.25^{ab} ± 1.25	97.50^{a} ± 0.94	98.75^{a} ± 0.82
Fl 3.0 µL	88.13^{ab} ± 3.26	94.38^{ab} ± 2.20	96.25^{ab} ± 1.83	97.50^{abc} ± 0.94
Fl 3.5 µL	83.75^{abc} ± 3.75	91.88^{ab} ± 1.88	94.38^{abc} ± 1.75	96.25^{abc} ± 1.25

Fl 4.0 µL	87.50^{abc} ± 3.78	93.13^{ab} ± 2.30	98.13^{a} ± 1.32	98.13^{ab} ± 1.32
Fl 4.5 µL	95.63^{a} ± 1.48	97.50^{a} ± 1.34	97.50^{a} ± 1.34	99.38^{a} ± 0.63
Fl 5.0 µL	92.50^{ab} ± 2.31	95.00^{ab} ± 2.11	96.25^{ab} ± 1.57	97.50^{abc} ± 1.34

Table S3. Canola seed germination flavonoid treatments (150 mM NaCl conditions).

Treatments	18 h ± SE	24 h ± SE	30 h ± SE	36 h ± SE
***p*-value**	< 0.0001	0.0001	0.0139	0.0110
Ctrl U.P (Unprimed)	8.75^{e} ± 2.06	70.63^{b} ±6.58	90.00^{bc} ± 2.50	91.88^{cd} ± 2.10
Ctrl P (Primed)	38.13^{d} ± 5.08	87.50^{a} ± 3.66	93.13^{ab} ± 2.10	93.75^{bcd} ± 1.83
Fl 0.5 µL	68.13^{bc} ± 10.09	88.13^{a} ± 5.66	94.38^{ab} ± 2.90	97.50^{ab} ± 1.34
Fl 1.0 µL	61.25^{c} ± 9.90	74.38^{b} ± 8.89	84.38^{c} ± 6.30	91.25^{d} ± 3.37
Fl 1.5 µL	74.38^{abc} ± 5.55	96.25^{a} ± 0.82	96.88^{ab} ± 0.91	98.13^{ab} ± 0.91
Fl 2.0 µL	80.63^{ab} ± 5.78	96.88^{a} ± 1.32	97.50^{a} ± 0.94	98.13^{ab} ± 0.91
Fl 2.5 µL	67.50^{bc} ± 10.52	92.50^{a} ± 3.54	96.88^{ab} ± 1.32	97.50^{ab} ± 1.34
Fl 3.0 µL	76.88^{abc} ± 4.43	93.13^{a} ± 2.66	95.63^{ab} ± 1.75	96.25^{abc} ± 1.83
Fl 3.5 µL	84.38^{ab} ± 3.95	93.75^{a} ± 2.27	97.50^{a} ± 2.50	98.75^{a} ± 1.25
Fl 4.0 µL	76.88^{abc} ± 3.40	93.75^{a} ± 2.27	96.25^{ab} ± 1.57	98.13^{ab} ± 1.32
Fl 4.5 µL	90.63^{a} ± 2.58	95.00^{a} ± 1.34	96.25^{ab} ± 1.25	98.13^{ab} ± 0.91
Fl 5.0 µL	83.13^{ab} ± 3.26	96.25^{a} ± 1.25	96.88^{ab} ± 1.32	97.50^{ab} ± 1.34

Table S4. Canola seed germination flavonoid treatments (175 mM NaCl conditions).

Treatments	18 h ± SE	24 h ± SE	30 h ± SE	36 h ± SE
***p*-value**	< 0.0001	0.0001	0.0001	0.0001
Ctrl U.P (Unprimed)	2.50^{e} ± 1.34	46.25^{b} ± 5.88	80.00^{bc} ± 4.33	88.13^{ab} ± 1.62
Ctrl P (Primed)	25.63^{cd} ± 5.86	80.63^{a} ± 2.40	88.75^{ab} ± 2.06	90.00^{a} ± 1.89
Fl 0.5 µL	22.50^{ed} ± 7.13	51.25^{b} ± 12.98	67.50^{c} ± 10.94	75.63^{bc} ± 8.99
Fl 1.0 µL	41.25^{bcd} ± 10.85	58.75^{b} ± 11.37	68.13^{c} ± 10.94	78.75^{bc} ± 8.54
Fl 1.5 µL	48.75^{ab} ± 10.08	90.63^{a} ± 1.48	94.38^{a} ± 1.48	96.25^{a} ± 1.25
Fl 2.0 µL	47.50^{ab} ± 9.11	81.25^{a} ± 2.80	96.25^{a} ± 1.83	97.50^{a} ± 0.94
Fl 2.5 µL	48.75^{ab} ± 11.01	83.13^{a} ± 5.26	96.88^{a} ± 1.32	98.13^{a} ± 0.91
Fl 3.0 µL	43.75^{ab} ± 8.49	83.13^{a} ± 3.65	93.13^{ab} ± 2.30	95.63^{a} ± 1.75
Fl 3.5 µL	49.38^{ab} ± 6.37	86.25^{a} ± 2.27	93.13^{ab} ± 1.88	95.00^{a} ± 1.34
Fl 4.0 µL	34.38^{bcd} ± 3.59	83.13^{a} ± 2.98	91.88^{ab} ± 1.88	95.63^{a} ± 1.75
Fl 4.5 µL	67.50^{a} ± 1.89	92.50^{a} ± 2.50	95.63^{a} ± 1.99	96.88^{a} ± 1.32
Fl 5.0 µL	65.63^{a} ± 3.05	93.75^{a} ± 1.57	96.88^{a} ± 1.62	97.50^{a} ± 1.34

Table S5. Canola seed germination flavonoid treatments (200 mM NaCl conditions).

Treatments	18 h ± SE	24 h ± SE	30 h ± SE	36 h ± SE
***p*-value**	**< 0.0001**	**< 0.0001**	**< 0.0001**	**< 0.0001**
Ctrl U.P (Unprimed)	0.63^{f} ± 0.63	15.63^{f} ± 4.38	53.13^{c} ± 10.85	76.88^{bc} ± 6.54
Ctrl P (Primed)	6.25^{ef} ± 2.27	64.38^{abcd} ± 4.06	76.88^{ab} ± 3.40	82.50^{abc} ± 4.01
Fl 0.5 µL	16.25^{cdef} ± 4.89	45.63^{de} ± 12.52	58.13^{bc} ± 14.20	66.88^{cd} ± 12.85
Fl 1.0 µL	15.00^{cdef} ± 4.43	38.75^{ef} ± 11.49	50.63^{c} ± 14.47	57.50^{d} ± 15.38
Fl 1.5 µL	13.13^{cdef} ± 3.26	54.38^{bcde} ± 7.59	80.63^{a} ± 3.46	95.63^{a} ± 1.75
Fl 2.0 µL	10.63^{def} ± 2.90	43.13^{de} ± 7.13	75.63^{ab} ± 6.37	89.38^{ab} ± 5.93
Fl 2.5 µL	20.63^{cde} ± 6.51	49.38^{cde} ± 13.07	83.75^{a} ± 3.98	93.75^{ab} ± 0.82
Fl 3.0 µL	27.50^{bcd} ± 3.54	77.50^{ab} ± 5.75	89.38^{a} ± 3.05	94.38^{ab} ± 1.13
Fl 3.5 µL	27.50^{bcd} ± 4.72	80.63^{a} ± 4.17	93.13^{a} ± 2.49	95.63^{a} ± 1.48
Fl 4.0 µL	30.00^{bc} ± 4.43	71.88^{abc} ± 6.68	86.88^{a} ± 4.81	94.38^{ab} ± 2.40
Fl 4.5 µL	42.50^{ab} ± 14.27	75.00^{ab} ± 9.45	90.63^{a} ± 3.46	96.25^{a} ± 1.83

Fl 5.0 µL	54.38^{a} ± 12.66	76.25^{ab} ± 7.95	91.25^{a} ± 3.24	95.63^{a} ± 1.99

Table S6: Soybean seed germination flavonoids (optimal conditions)

**Treatments**	**24 h ± SE**	**48 h ± SE**	**72 h ± SE**
***p*-value**	0.1076	0.6634	0.4130
Ctrl P (Primed)	83.75^{ab} ± 3.24	96.25^{a} ± 1.83	98.75^{a} ± 1.25
Ctrl U.P (Unprimed)	77.50^{b} ± 2.50	90.00^{a} ± 2.63	92.50^{ab} ± 1.64
Fl 1.0 µL	90.00^{a} ± 3.78	93.75^{a} ± 2.63	93.75^{ab} ± 2.63
Fl 2.0 µL	90.00^{a} ± 3.78	95.00^{a} ± 2.67	95.00^{ab} ± 2.67
Fl 3.0 µL	80.00^{ab} ± 3.78	91.25^{a} ± 2.95	91.25^{b} ± 2.95
Fl 4.0 µL	85.00^{ab} ± 3.78	92.50^{a} ± 2.50	93.75^{ab} ± 1.83
Fl 5.0 µL	87.50^{ab} ± 3.66	91.25^{a} ± 3.50	92.50^{ab} ± 3.13

Table S7. Soybean seed germination flavonoids (80 mM NaCl conditions).

**Treatments**	**24 h ± SE**	**48 h ± SE**	**72 h ± SE**
***p*-value**	< 0.0001	0.0513	0.1486
Ctrl P (Primed)	38.75^{b} ± 3.98	87.50^{ab} ± 2.50	91.25^{ab} ± 2.27
Ctrl U.P (Unprimed)	16.25^{c} ± 3.75	82.50^{b} ± 3.66	87.50^{b} ± 3.13
Fl 1.0 µL	75.00^{a} ± 3.78	95.00^{a} ± 1.89	96.25^{a} ± 1.83
Fl 2.0 µL	70.00^{a} ± 4.63	86.25^{b} ± 4.20	91.25^{ab} ± 3.98
Fl 3.0 µL	80.00^{a} ± 2.67	88.75^{ab} ± 2.27	88.75^{ab} ± 2.27
Fl 4.0 µL	75.00^{a} ± 6.27	95.00^{a} ± 3.27	96.25^{a} ± 2.63
Fl 5.0 µL	80.00^{a} ± 2.67	90.00^{ab} ± 2.67	95.00^{ab} ± 2.67

Table S8. Soybean seed germination flavonoids (100 mM NaCl conditions).

**Treatments**	**24 h ± SE**	**48 h ± SE**	**72 h ± SE**
***p*-value**	< 0.0001	0.0787	0.2463
Ctrl P (Primed)	27.50^{b} ± 2.50	86.25^{ab} ± 4.60	90.00^{ab} ± 4.23
Ctrl U.P (Unprimed)	15.00^{c} ± 5.00	80.00^{b} ± 4.23	83.75^{b} ± 4.60
Fl 1.0 µL	63.75^{a} ± 5.65	91.25^{a} ± 3.50	92.50^{ab} ± 2.50
Fl 2.0 µL	61.25^{a} ± 4.79	87.50^{ab} ± 3.66	88.75^{ab} ± 2.95
Fl 3.0 µL	70.00^{a} ± 3.27	93.75^{a} ± 2.63	93.75^{a} ± 2.63
Fl 4.0 µL	72.50^{a} ± 2.50	91.25^{a} ± 2.27	93.75^{a} ± 1.83
Fl 5.0 µL	68.75^{a} ± 3.50	93.75^{a} ± 2.63	93.75^{a} ± 2.63

Table S9. Soybean seed germination flavonoids (120 mM NaCl conditions).

**Treatments**	**24 h ± SE**	**48 h ± SE**	**72 h ± SE**
***p*-value**	< 0.0001	< 0.0001	0.0309
Ctrl P (Primed)	11.25^{b} ± 3.50	86.25^{a} ± 3.75	93.75^{a} ± 2.63
Ctrl U.P (Unprimed)	0.00^{b} ± 0.00	43.75^{b} ± 14.38	83.75^{b} ± 3.75
Fl 1.0 µL	46.25^{a} ± 4.60	92.50^{a} ± 3.13	93.75^{a} ± 2.63
Fl 2.0 µL	58.75^{a} ± 6.11	93.75^{a} ± 3.24	97.50^{a} ± 2.50
Fl 3.0 µL	56.25^{a} ± 5.96	93.75^{a} ± 2.63	97.50^{a} ± 1.64
Fl 4.0 µL	48.75^{a} ± 5.81	87.50^{a} ± 4.12	91.25^{ab} ± 2.95
Fl 5.0 µL	50.00^{a} ± 4.23	90.00^{a} ± 5.67	93.75^{a} ± 3.75

CFS priming and seed germination of canola and soybean (Tables)

The data in Tables S10 to S13 represent the mean percentage of 8 replications ± standard error. Different letters indicate values determined by Tukey's multiple mean comparison to be significantly different ($p < 0.05$) among treatments.

Table S10. Canola seed germination CFS (optimal conditions).

Treatments	18 h ± SE	24 h ± SE	30 h ± SE	36 h ± SE
***p*-value**	0.0102	0.8318 (n.s)	0.7877 (n.s)	0.7349 (n.s)
Ctrl U.P (Unprimed)	85.00^{b} ± 3.54	93.75 ± 2.06	95.00 ± 2.31	95.00 ± 2.31
Ctrl P (Primed)	90.00^{ab} ± 2.67	94.38 ± 1.13	95.00 ± 0.94	95.63 ± 1.13
CFS (1:50)	94.38^{a} ± 1.75	95.63 ± 1.13	96.88 ± 0.91	97.50 ± 0.94
CFS (1:100)	95.63^{a} ± 0.63	96.25 ± 0.82	96.25 ± 0.82	96.88 ± 0.91
CFS (1:250)	95.63^{a} ± 1.75	95.63 ± 1.75	96.25 ± 1.83	96.88 ± 1.88
CFS (1:500)	95.00^{a} ± 1.53	96.88 ± 1.51	97.50 ± 1.25	98.13 ± 0.86
CFS (1:1000)	94.38^{a} ± 2.40	95.63 ± 1.99	97.50 ± 1.34	97.50 ± 1.34

Table S11. Canola seed germination CFS (150 mM NaCl conditions).

Treatments	18 h ± SE	24 h ± SE	30h ± SE	36 h ± SE
***p*-value**	< 0.0001	0.0804	0.6032	0.0549
Ctrl U.P (Unprimed)	13.13^{d} ± 0.91	82.50^{c} ± 3.13	91.88^{a} ± 2.66	91.88^{c} ± 2.66
Ctrl P (Primed)	38.13^{c} ± 5.08	87.50^{abc} ± 3.66	93.13^{a} ± 2.10	93.75^{bc} ± 1.83
CFS (1:50)	53.75^{b} ± 3.98	88.75^{abc} ± 3.24	93.75^{a} ± 1.57	95.00^{abc} ± 0.94
CFS (1:100)	55.00^{b} ± 6.20	86.25^{bc} ± 3.75	93.75^{a} ± 1.25	95.63^{abc} ± 0.63
CFS (1:250)	80.00^{a} ± 6.55	91.25^{ab} ± 1.57	94.38^{a} ± 1.13	95.00^{abc} ± 0.94
CFS (1:500)	52.50^{cb} ± 5.86	95.63^{a} ± 1.38	96.88^{a} ± 0.86	98.75^{a} ± 0.77
CFS (1:1000)	53.13^{cb} ± 5.66	89.38^{abc} ± 2.40	93.13^{a} ± 2.10	96.88^{ab} ± 1.32

Table S12. Soybean seed germination CFS (optimal conditions).

Treatments	24 h ± SE	48 h ± SE	72 h ± SE
***p*-value**	0.0004	0.0005	0.0042
Ctrl P (Primed)	83.75^{ab} ± 3.24	96.25^{ab} ± 1.83	98.75^{ab} ± 1.25
Ctrl U.P (Unprimed)	77.50^{bc} ± 2.50	90.00^{bc} ± 2.67	92.50^{bc} ± 1.64
CFS (1:50)	70.00^{c} ± 4.63	87.50^{c} ± 2.50	92.50^{bc} ± 3.13
CFS (1:100)	70.00^{c} ± 3.78	85.00^{c} ± 4.23	86.25^{c} ± 3.75
CFS (1:250)	76.25^{bc} ± 2.63	95.00^{ab} ± 2.67	92.50^{bc} ± 3.13
CFS (1:500)	75.00^{bc} ± 4.63	97.50^{a} ± 1.64	96.25^{ab} ± 1.83
CFS (1:1000)	92.50^{a} ± 2.50	100.00^{a} ± 0.00	100.00^{a} ± 0.00

Table S13. Soybean seed germination CFS (100 mM NaCl conditions).

Treatments	24 h ± SE	48 h ± SE	72 h ± SE
***p*-value**	0.0042	0.0237	0.1231
Ctrl P (Primed)	27.50abcd ± 2.50	86.25 ± 4.60	90.00 ± 4.23
Ctrl U.P (Unprimed)	15.00d ± 5.00	80.00 ± 4.23	83.75 ± 4.60
CFS (1:50)	17.50cd ± 4.53	65.00 ± 5.35	77.50 ± 3.66
CFS (1:100)	31.25abc ± 4.79	86.25 ± 4.20	90.00 ± 3.78
CFS (1:250)	23.75bcd ± 7.54	80.00 ± 6.55	90.00 ± 2.67
CFS (1:500)	42.50a ± 6.48	87.50 ± 3.66	91.25 ± 3.50
CFS (1:1000)	38.75ab ± 5.15	85.00 ± 4.23	90.00 ± 3.78

Priming of flavonoids and CFS (combined) on seed germination of canola and soybean (Tables)

The data in Tables S14 to S17 represent the mean percentage of 8 replications ± standard error. Different letters indicate values determined by Tukey's multiple mean comparison to be significantly different ($p < 0.05$) among treatments.

Table S14. Canola seed germination CFS and flavonoids (combined) (optimal conditions).

Treatments	18 h ± SE	24 h ± SE	30 h ± SE	36 h ± SE
p-value	0.0035	0.6503	0.4006	0.4130
Ctrl U.P (Unprimed)	85.00[c] ± 2.83	93.75[a] ± 2.27	95.00[b] ± 1.89	95.63[a] ± 1.48
Ctrl P (Primed)	90.00[bc] ± 2.67	94.38[a] ± 1.13	95.00[b] ± 0.94	95.63[a] ± 1.13
Fl 4.5 + CFS 1:50	94.38[ab] ± 2.40	95.63[a] ± 2.20	96.88[ab] ± 1.62	96.88[a] ± 1.62
Fl 4.5 + CFS 1:100	93.13[ab] ± 1.62	95.63[a] ± 1.48	96.25[ab] ± 1.25	96.25[a] ± 1.25
Fl 4.5 + CFS 1:250	95.63[ab] ± 1.75	95.63[a] ± 1.75	96.88[ab] ± 1.32	97.50[a] ± 0.94
Fl 4.5 +CFS 1:500	90.63[ab] ± 1.06	95.00[a] ± 1.25	97.50[ab] ± 0.88	98.13[a] ± 0.86
Fl 4.5 + CFS 1:1000	96.25[a] ± 0.82	98.13[a] ± 0.91	98.75[a] ± 0.82	98.75[a] ± 0.82

Table S15. Canola seed germination CFS and flavonoids (combined) (150 mM NaCl conditions).

Treatments	18 h ± SE	24 h ± SE	30 h ± SE	36 h ± SE
p-value	0.0001	0.3501	0.7101	0.0540
Ctrl U.P (Unprimed)	13.13[e] ± 0.91	82.50[b] ± 3.13	91.88[a] ± 2.66	91.88[c] ± 2.66
Ctrl P (Primed)	38.13[d] ± 5.08	87.50[ab] ± 3.66	93.13[a] ± 2.10	93.75[abc] ± 1.83
Fl 4.5 + CFS 1:50	51.25[c] ± 2.80	89.38[ab] ± 1.75	93.75[a] ± 1.25	98.13[ab] ± 0.91
Fl 4.5 + CFS 1:100	61.88[bc] ± 4.43	90.00[ab] ± 2.50	94.38[a] ± 1.75	95.00[abc] ± 1.34
Fl 4.5 + CFS 1:250	86.88[a] ± 4.00	90.63[a] ± 1.48	95.00[a] ± 1.34	97.50[ab] ± 0.94
Fl 4.5 +CFS 1:500	57.50[bc] ± 3.31	86.88[ab] ± 2.92	91.25[a] ± 2.61	93.13[bc] ± 2.64
Fl 4.5 + CFS 1:1000	66.25[b] ± 3.98	90.63[a] ± 2.74	95.63[a] ± 1.75	98.75[a] ± 0.82

Table S16. Soybean seed germination flavonoids and CFS (Combined) (optimal conditions).

Treatments	24 h ± SE	48 h ± SE	72 h ± SE
p-value	0.0014	0.4283	0.0829
Ctrl P (Primed)	83.75[ab] ± 3.24	90.00[a] ± 2.67	92.50[bc] ± 1.64
Ctrl U.P (Unprimed)	77.50[ab] ± 2.50	96.25[a] ± 1.83	98.75[a] ± 1.25
Fl 1.0 + CFS 1:50	62.50[c] ± 4.12	91.25[a] ± 2.95	93.75[abc] ± 2.63
Fl 1.0 + CFS 1:100	73.75[b] ± 3.75	92.50[a] ± 2.50	91.25[c] ± 2.27
Fl 1.0 + CFS 1:250	78.75[ab] ± 5.15	92.50[a] ± 2.50	92.50[bc] ± 2.50
Fl 1.0 +CFS 1:500	82.50[ab] ± 3.66	96.25[a] ± 1.83	97.50[ab] ± 1.64
Fl 1.0 + CFS 1:1000	87.50[a] ± 4.53	95.00[a] ± 2.67	96.25[abc] ± 1.83

Table S17. Soybean seed germination flavonoids and CFS (Combined) (100 mM NaCl conditions).

Treatments	24 h ± SE	48 h ± SE	72 h ± SE
p-value	< 0.0001	0.5190	0.3262
Ctrl P (Primed)	27.50[cd] ± 2.50	86.25[a] ± 4.60	90.00[a] ± 4.23
Ctrl U.P (Unprimed)	15.00[de] ± 5.00	80.00[a] ± 4.23	83.75[a] ± 4.60
Fl 1.0 + CFS 1:50	12.50[e] ± 3.13	80.00[a] ± 4.63	83.75[a] ± 3.75
Fl 1.0 + CFS 1:100	42.50[ab] ± 6.48	85.00[a] ± 4.23	91.25[a] ± 2.95
Fl 1.0 + CFS 1:250	36.25[bc] ± 7.06	81.25[a] ± 5.15	83.75[a] ± 5.65
Fl 1.0 +CFS 1:500	41.25[bc] ± 6.11	78.75[a] ± 6.93	85.00[a] ± 5.00
Fl 1.0 + CFS 1:1000	56.25[a] ± 4.60	91.25[a] ± 2.95	95.00[a] ± 1.89
